# Comparison of gastrointestinal symptoms and findings in renal replacement therapy modalities

**DOI:** 10.1186/s12882-022-02893-6

**Published:** 2022-07-23

**Authors:** Doğu Karahan, İdris Şahin

**Affiliations:** 1 Departmant of Internal Medicine, Malatya Turgut Özal University School of Medicine, Malatya Training and Research Hospital, Özalper. Turgut Özal Ave. No:4, 44330 Malatya, Turkey; 2 Departmant of Nephrology, İnonu University School of Medicine, Malatya, Turkey

**Keywords:** Chronic kidney disease, Gastrointestinal symptoms, Hemodialysis, Peritoneal dialysis

## Abstract

**Objective:**

Chronic kidney disease (CKD) affects gastrointestinal system (GIS) and causes histological, functional and mucosal changes. There are scarce data investigating GIS symptoms and findings in patients with CKD stage III-V, receiving hemodialysis (HD) and peritoneal dialysis (PD). In this study, we aimed to evaluate the frequency of gastrointestinal symptoms and findings and compare between renal replacement therapies.

**Method:**

A total of 290 patients (97 in CKD stage III-V, 92 PD, 101 HD) were included in this study. Gastrointestinal complaints, diseases, background characteristics of patients and drugs they used were questioned by interviews, forms were filled and examinations of patients were performed. Results of upper GIS endoscopy, colonoscopy, abdominal ultrasonography and tomography of patients were evaluated.

**Results:**

The most common signs were dyspepsia (50%), nausea (45%) and epigastric pain (44%) among all patients, generally. Gastrointestinal disorders like gastritis (62%) and gastroesophageal reflux (39%) were frequent in patients. Prevalence of patients with weight loss was 20% in predialysis and 8% in PD and the ratio was higher in predialysis group statistically significantly (*p* = 0,016). The prevalence of gastritis was 70% in PD, 55% in HD and the prevalence of hemorrhoids was 24% in PD and 12% in HD. The prevalence of gastritis and hemorrhoids was higher in the PD group than in the HD group statistically significantly (*p* = 0.043, *p* = 0.028), otherwise, there wasn’t a difference between the PD and predialysis groups, statistically significantly.

**Conclusion:**

This study showed that; gastrointestinal symptoms and disorders were very common in CKD, besides this; while gastritis and hemorrhoids were more frequent in the PD, esophagitis and hiatal hernia were more frequent in the HD.

**Supplementary Information:**

The online version contains supplementary material available at 10.1186/s12882-022-02893-6.

## Introduction

Chronic kidney disease (CKD) is a pathophysiological process with many etiological causes, often leading to end-stage renal disease (ESRD), with a progressive and irreversible decrease in the number of nephrons and kidney functions. Uremia is a clinical and laboratory syndrome, that occurs because of renal failure and reflects dysfunction of all affected organs, including the gastrointestinal system (GIS) [1].

Gastrointestinal symptoms and signs are quite common in patients with CKD. Studies have found that the frequency of symptoms is around 77–79% [2]. Symptoms and findings are related to all departments from the upper gastrointestinal tract to the lower gastrointestinal tract and can be divided into 3 categories as membrane problems, functional problems and histopathological problems. Membrane problems such as glossitis, stomatitis, esophagitis, enteritis, colitis, ileitis; functional problems such as loss of appetite, nausea, vomiting, hematemesis, constipation, diarrhea, abdominal distention, reflux, ileus; histopathological changes such as ulcers, bleeding and pancreatitis may be observed [3]. Complications like an increase in infectious diseases such as HBV, HCV, CMV, peritonitis, increased incidence of cancer, treatment-related gastrointestinal bleeding, pancreatitis, cholelithiasis and increased incidence of hernia are also frequently encountered in patients with CKD [3–5].

There are many causes of GIS symptoms and signs seen in CKD patients, but the underlying mechanisms in its formation have not been fully elucidated. Some studies suggest that some common complaints such as nausea, vomiting, dyspepsia, bloating and constipation may be associated with delayed gastric emptying and impaired gastric myoelectric activity [6].

However, the changes in GIS also show differences according to the stage of CKD and the type of renal replacement therapy (RRT) applied. Again, it is not clear whether the underlying causes of GI involvement in chronic kidney disease depend on uremia or the type of treatment administered [2]. However, identifying CKD patients with gastrointestinal tract symptoms and signs and treating these patients with recommendations and medications are important in terms of improving gastrointestinal quality of life [7]..

In this study, we aimed to determine the frequency of GIS symptoms and signs in patients with CKD receiving conservative treatment or hemodialysis and peritoneal dialysis and also to compare the diversity of symptoms and signs in these patients who received different renal replacement therapy modalities.

## Materials and methods

This study was carried out at Inonu University Faculty of Medicine Turgut Ozal Medical Center Internal Medicine and Nephrology Clinics. Patients who were followed up with stage III-V CKD in the predialysis period in the nephrology clinic, patients receiving continuous ambulatory peritoneal dialysis treatment (CAPD) in the Peritoneal Dialysis Unit and cases who underwent hemodialysis in the Inonu University Turgut Ozal Medical Center Hemodialysis Unit were included in the study. The study was carried out in a single center for a period of 36 months. 290 patients agreed to participate in the study voluntarily. Patients older than 18 years were included in the study. While forming the groups, the age and gender distribution were designed to be balanced. While the cases were selected in the predialysis group, the cases followed up in our clinic with the diagnosis of CKD for at least 6 months were included in the study.

The ethics committee approval was received for this study from the Local Research Ethics Committee of Inonu University (2008/0118). Informed consent forms were signed by the patients. Cases younger than 18 years of age, cases with acute renal failure and patients followed up with stage I-II CKD were excluded from the study. Patients with communication problems and those with poor cooperation and orientation were not included in the study.

97 patients followed up with CKD stage III-V, 101 patients on hemodialysis and 92 patients on peritoneal dialysis were included in the study. The patients were asked about their gastrointestinal system complaints by face-to-face interview method. Questionnaire forms of the patients were filled during their routine examinations and sessions. GIS diseases and operations in the past were questioned. The history of the patients, the drugs used and the results of previous endoscopy, colonoscopy, abdominal ultrasonography and abdominal tomography were recorded. No new endoscopy, colonoscopy, ultrasonography and abdominal tomography were requested to the patients. Detailed physical examinations of all patients were performed.

The complaints of epigastric pain-burning, abdominal pain, nausea, vomiting, anorexia, chronic diarrhea, constipation, dysphagia, odynophagia, burping, regurgitation, heartburn, dyspepsia, dry cough, weight loss, hematemesis, melena, hematochezia, pain in the anal region, anal itching, rectal fresh bleeding, incontinence and icterus were questioned and evaluated.

The complaints, findings and existing GIS diseases of the patients in the last 6 months were questioned and compared. The causes and the duration of CKD, the type and duration of RRT, the drugs used and the history of the patients were recorded. The imaging methods applied to the cases in the last 6 months were scanned from the system. In the retrospective scan, the results of upper GIS endoscopy in 45 patients, colonoscopy in 13 patients, abdominal ultrasonography in 95 patients and abdominal tomography in 44 patients were obtained.

Blood samples for necessary laboratory investigations were taken from all patients after 12 hours of fasting. Blood collection was performed before routine hemodialysis seance in hemodialysis patients. Samples from the patients in the predialysis and PD groups were taken on an empty stomach in the morning during their routine controls. Blood samples taken from the antecubital vein were put into different tubes for the purpose of complete blood count, biochemical, hormonal and serological evaluation. Samples taken without anticoagulant for biochemical analyses were incubated at 37 °C for 20 minutes and then turned at 2500 rpm for 5 minutes and serums were obtained. After separating the serums for biochemical and hormonal analyses, blood urea nitrogen (BUN), creatinine, uric acid, total protein, albumin, total cholesterol, triglyceride, high-density cholesterol (HDL- cholesterol), calcium (Ca), phosphorus (P) levels in all patients were worked automatically in an Abbott Aeroset (Sentinel Diagnostic, Milano, Italy) autoanalyzer with Abbott brand (Sentinel Diagnostic Milano, Italy) commercial kits.

The VLDL values of the patients were found with the formula VLDL = cholesterol/5. LDL values were measured with the LDL = total cholesterol-(total cholesterol/5 + HDL) formula (Friedwald formula).

Serum PTH (parathormone) levels of the patients were studied with the radioimmunoassay (RIA) method using the IMMULITE 2000 (Siemens Medical Solutions Diagnostics, Los Angeles, CA, USA) device with commercial kits from the Immulyte brand. Ferritin level was studied with a Dade Boehring brand BN 2 model (Siemens Healthcare Diagnostic Products Gmbh, Marburg, Germany) device. The unit was pg/ml for PTH and ng/ml for ferritin.

The complete blood count of the patients was automatically performed with the Couter LYSE S Diff-lytic Reagent kit on the BeckmanCoulter LH 780 Analyzer (Beckman, USA) device from 2 ccs of blood collected in standard EDTA tubes in the Hematology Laboratory of the Inonu University Faculty of Medicine.

The blood samples taken from the patients for HBsAg, Anti-HBs, and Anti-HCV were studied with the Diapro (Dia. Pro Diagnostic Broprobes, Milano Italy) kit in the Ali Rad Micro-Elisa device at the Microbiology Laboratory of Inonu University.

### Statistical procedures

Numerical data were presented as mean ± standard deviation and percentage ratio. The Chi-square test and independent t-test were used for statistical comparisons. ANOVA (analysis of variants) test was used for comparison between groups. Statistical comparisons were made using a computer program called SPSS (Statistical Package for Social Science) for Windows vs 11.0. *P* < 0.05 was considered statistically significant.

## Results

A total of 290 patients (97 patients with CKD stage III-V, 101 patients on hemodialysis and 92 patients on peritoneal dialysis) were included in the study. There were 168 males and 122 females. The mean age of the patients was 50.4 ± 15.4 (18–85) years. The mean age of patients undergoing hemodialysis was 51.7 ± 15.7; the mean age of the patients treated with CAPD was 45.4 ± 13.2 years and the mean age of the patients followed up CKD stage III-V was 54.7 ± 15.9 years. The mean duration of CKD in patients included in the study was 55.6 ± 58.6 months. The mean RRT time was 38.5 ± 44.5 months, the mean hemodialysis time was 23.8 ± 42.4 months and the mean peritoneal dialysis time was 17.9 ± 28.9 months.

**Fig. 1:**
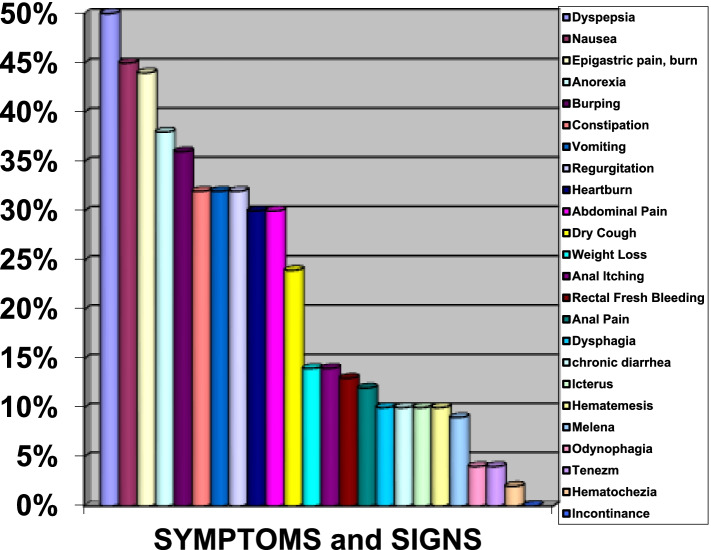
Percentage of distribution of gastrointestinal system complaints and signs in patients with chronic kidney disease

**Fig. 2:**
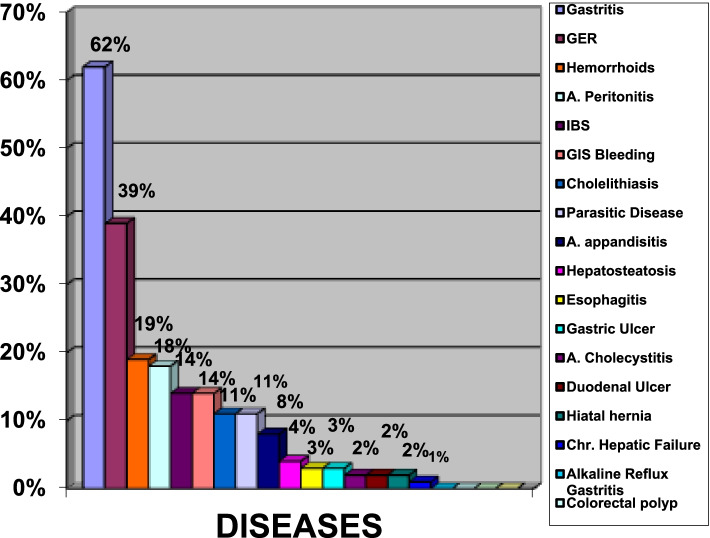
Percentage of distribution of gastrointestinal system diseases in patients with chronic kidney disease

When all patients were evaluated, the most common GIS complaints and signs were being lined up as dyspepsia in 50%, nausea in 45%, epigastric pain and burning in 44%, anorexia in 38%, burping in 36%, constipation, vomiting and regurgitation in 32%, abdominal pain and heartburn in 30%. Of the 290 patients included in the study, 62% had gastritis findings, 14% had irritable bowel disease (IBS) findings, 19% had hemorrhoids, 39% had gastroesophageal reflux findings, 11% had a parasitic disease, 11% had cholelithiasis and 14% had gastrointestinal bleeding. Concomitant chronic liver disease was detected in two patients. Inflammatory polyps were detected in one patient’s recto sigmoidoscopy. Six patients had an attack of acute cholecystitis, one patient was endoscopically diagnosed with alkaline reflux gastritis (in the hemodialysis group). There were gastric ulcers in 8 patients, esophagitis in 10 patients, duodenal ulcers in 5 patients and hiatal hernia in 5 patients diagnosed endoscopically. Barrett’s esophagus was diagnosed pathologically in one patient who was treated for CAPD. Hepatosteatosis was present in 12 of 95 patients who has abdominal ultrasonography results. 50% of the patients receiving peritoneal dialysis treatment had at least one episode of acute peritonitis. The results are shown in Fig. 1 and Fig. 2.

Gastrointestinal system complaints and findings of the patients were compared between predialysis-hemodialysis, predialysis-peritoneal dialysis and hemodialysis-peritoneal dialysis patient groups.

Epigastric pain and burning were present in 45% of both hemodialysis and peritoneal dialysis patients, and 42% of predialysis patients and there was no difference between the groups. Abdominal pain was detected as 29% in the HD group, 37% in the PD group, 26% in the predialysis group and there was no statistically significant difference between the groups. However, abdominal pain was higher in the PD group than in the other groups. Nausea was present 45% in the HD group, 39% in the PD group, 53% in the predialysis group; vomiting was present in 35% of HD patients, 32% of PD patients, 33% of predialysis patients and no statistical difference were found between the groups in terms of these complaints. The results are shown in Table 1.

**Table 1:** The prevalence of gastrointestinal complaints in predialysis, hemodialysis and peritoneal dialysis patient groups

COMPLAINTS AND SIGNS	Predialysis percentage *n* = 97	Hemodialysis percentage *n* = 101	Peritoneal dialysis percentage *n* = 92
Dyspepsia	45%	49%	55%
Nausea	53%	45%	39%
Epigastric pain-burning	42%	45%	45%
Anorexia	36%	37%	42%
Burping	33%	31% * (*p* = 0,032)	46% * (*p* = 0,032)
Constipation	32%	28%	35%
Vomiting	33%	35%	32%
Regurgitation	33%	27%	36%
Heartburn	30%	27%	33%
Abdominal Pain	26%	29%	37%
Dry Cough	24%	21%	28%
Weight Loss	20% ** (*p* = 0,016)	16%	8% **(*p* = 0,016)
Anal Itching	9%	14%	20%
Rectal Fresh Bleeding	11%	12%	14%
Anal Pain	6%	15%	12%
Dysphagia	9%	13%	8%
Chronic Diarrhea	10%	11%	8%
Icterus	9%	11%	10%
Hematemesis	6%	14%	11%
Melena	4%	12%	12%
Odynophagia	2%	5%	3%
Tenezm	5%	3%	1%
Hematochezia	2%	4%	_
Incontinence	_	1%	_
Patients with no symptoms	8%	9%	9%

* *p* = 0.032: Belching is significantly higher in the PD group than in the HD group

** *p* = 0.016: Weight loss is significantly higher in the predialysis group than in the PD group

Although dyspepsia, regurgitation, heartburn and dry cough were more common in the peritoneal dialysis group than in HD and predialysis groups, there was no statistically significant difference between the groups. Weight loss was 20% in predialysis, 16% in hemodialysis and 8% in peritoneal dialysis. There was a statistically significant difference between the predialysis patients and the peritoneal dialysis patient group in terms of weight loss and it was statistically significantly higher in the predialysis patient group (*p* = 0.016). Although weight loss was more common in the HD group, no statistically significant difference was found between the HD and PD groups. Again, no statistically significant difference was found between the groups in terms of dysphagia and odynophagia complaints. Burping was detected in 46% of PD patients, 31% of HD patients, 33% of predialysis patients and the rate was statistically significantly higher in the PD group than in the HD group (*p* = 0.032), but the difference between PD and predialysis was not statistically significant (*p* = 0.075).

When evaluated in terms of gastrointestinal findings and diseases, gastritis was present 70% in the PD group, 55% in the HD group; hemorrhoids were present at a prevalence of 24% in the PD group and 12% in the HD group; and the prevalence for both diseases was statistically significantly higher in the PD group than in the HD group (*p* = 0.043, *p* = 0.028, respectively). Gastritis was present 60% in the predialysis patient group and hemorrhoids were 21% and there was no statistically significant difference between the predialysis and the other groups in terms of these diseases. On the other hand, esophagitis was detected as 8% in the HD group, 2% in the PD group and 0% in the predialysis group. While the difference was statistically significantly higher in the hemodialysis group than in the predialysis group (*p* = 0.004), it was not statistically significant between the hemodialysis-peritoneal dialysis and the predialysis-peritoneal dialysis groups. Endoscopically diagnosed hiatal hernia was detected as 5% in the HD group and 0% in the PD group and the predialysis group. And the difference between HD-PD and HD-predialysis was evaluated as statistically significant (*p* = 0.025, *p* = 0.026, respectively).

Gastroesophageal reflux (GER) symptoms were detected in 34% of HD patients, 38% of predialysis patients and 45% of PD patients. Although GER symptoms were higher in the PD group than in the other groups, there was no statistically significant difference between the groups. The results are shown in Table 2.

**Table 2:** Percentage distribution of gastrointestinal diseases detected in predialysis, hemodialysis and peritoneal dialysis patient groups

GASTROINTESTINAL DISEASE	Predialysis percentage *n* = 97	Hemodialysis percentage *n* = 101	Peritoneal dialysis percentage *n* = 92
Gastritis	60%	55% * (*p* = 0,043)	70% * (*p* = 0,043)
GER	38%	34%	45%
Hemorrhoids	21%	12% ** (*p* = 0,028)	24% ** (*p* = 0,028)
A. Peritonitis	_	7%	50%
IBS	16%	11%	12%
GIS Bleeding	9%	18%	14%
Cholelithiasis	13%	11%	10%
Parasitic Disease	14%	11%	8%
A. Appendicitis	9%	6%	8%
Hepatosteatosis	5%	4%	3%
Esophagitis	_***(*p* = 0,004)	8% ***(*p* = 0,004)	2%
Gastric Ulcer	4%	2%	1%
A. Cholecystitis	2%	2%	2%
Duodenal Ulcer	2%	3%	_
Hiatal Hernia	_β (*p* = 0,026)	5% α(*p* = 0,025), β (*p* = 0,026)	_α (*p* = 0,025)
Chr. Hepatic Failure	1%	1%	_
Colorectal polyp	1%	_	_
Alkaline reflux gastritis	_	1%	_
A. Pancreatitis	_	1%	_
Ileus operation	_	_	1%

* *p* = 0,043: Gastritis is significantly higher in the PD group than in the HD group

** *p* = 0,028: Hemorrhoids are significantly higher in the PD group than in the HD group

*** *p* = 0,004: Esophagitis is significantly higher in the HD group than in the predialysis group

α *p* = 0,025: Hiatal hernia is significantly higher in the HD group than in the PD group

β *p* = 0,026: Hiatal hernia is significantly higher in the HD group than in the predialysis group

A 4% of all cases included in the study had HBsAg positivity and 3% had anti-HCV positivity.

Table 2 shows that 7% of the patients receiving hemodialysis treatment have a history of acute peritonitis. Some of the patients who entered hemodialysis in our clinic had previously received peritoneal dialysis treatment and switched to hemodialysis treatment for various reasons. Acute peritonitis positivity of 7% is due to the peritonitis experienced by these patients during the peritoneal dialysis treatment.

**Fig. 3:**
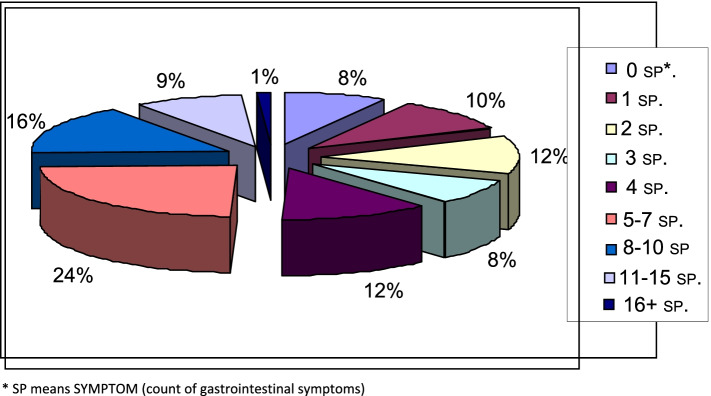
% Distribution of patients with CKD by the total number of gastrointestinal system symptoms. * SP means SYMPTOM (count of gastrointestinal symptoms)

Patients were also evaluated according to the number of symptoms and the number of symptoms was compared between the groups. 91.7% (*n* = 266) of the patients had at least one GIS-related symptom. The mean number of symptoms was calculated as 3.7 ± 2.2 in the HD group, 3.9 ± 2.2 in the PD group and 4.1 ± 2.1 in the predialysis group. There was no statistically significant difference about the number of symptoms in the comparison between the groups. The number of GIS symptoms did not change with the duration of CKD and RRT and there was no correlation between them. Symptom frequency rates are given in Fig. 3.

## Discussion

Gastrointestinal system symptoms and signs are frequently encountered in patients followed up with a diagnosis of CKD at all stages, from low clearance to those who underwent end-stage renal disease (ESRD) and RRT [8]. Some previous studies have reported that gastrointestinal symptoms are seen at a frequency of 32–79% in patients undergoing dialysis [9, 10]. Our study showed that more than 90% of patients with CKD had GIS symptoms. In addition, patients in all groups had an average of four GIS symptoms. These findings indicate that the gastrointestinal system is frequently affected in CKD, regardless of the type of treatment modalities.

Gastrointestinal complaints are common in uremic patients. In the studies, it was determined that GIS symptoms resulting from impaired gastric myoelectric activity, gastric hypomotility and prolonged gastric emptying were found to be especially frequent in the predialysis period in end-stage renal disease patients. It has been reported that this situation is associated with uremia [11]. However, the answer to the question of whether the impaired gastric motor function improves with dialysis treatment is not clear. Studies have evaluated whether the frequency of these complaints changes with the initiation of renal replacement therapy or changes according to the form of RRT. However, these studies give conflicting results. Schoonjans et al. [12] in their study in which they compared dyspeptic symptoms and gastric emptying times in patient groups receiving different renal replacement therapy; they found the prevalence of dysmotility-like dyspepsia to be the highest in peritoneal dialysis patients with 67.9%, then 53.6% in the predialysis group and 33.3% (*p* < 0.01) in the hemodialysis group (the difference was not statistically significant). Hiroshi et al. [11] reported that gastric motility improved and GIS symptoms decreased with HD treatment in their study in which they compared patients in the predialysis uremic period with those under hemodialysis treatment in terms of GIS symptoms and gastric motility. In a previous study done by Soffer et al. [13] comparing hemodialysis patients and normal subjects not on hemodialysis, no difference was found between hemodialysis patients and normal subjects in terms of gastric emptying time. In a later study by Van Vlem B et al., it was found that the gastric emptying time of patients with dyspeptic complaints in hemodialysis patients was significantly impaired compared to asymptomatic patients and normal individuals [10]. Kosmadakis et al. [14] reported in their study that gastric emptying is impaired in PD patients, regardless of the composition of dialysate and even when tested with an empty peritoneal cavity. In our study, although dyspeptic signs were the highest in the PD group, no statistically significant difference was found compared to the HD and predialysis groups. Our results are similar to literature data.

In our study, uremic symptoms such as nausea, vomiting, anorexia, constipation, weight loss and dyspepsia were compared between the predialysis, HD and PD treatment groups, and no statistically significant difference was found between the three groups. Although these symptoms were associated with uremia and impaired gastric emptying time, they did not differ according to the treatment modality in our study. When we evaluated weight loss in particular, the prevalence was found to be statistically significantly higher in the predialysis group than in the PD group, but no significant difference was found between the predialysis and HD groups in our study. Weight loss is considered among the findings associated with uremia and is considered as one of the indicators of the need for renal replacement therapy. However, when evaluated together with other uremic symptoms and findings, no significant difference was found between predialysis, PD and HD groups in our study. In this case, the mentioned symptoms cannot be explained only by uremia; this suggests that it may be due to other factors that have not yet been identified beside the delay in gastric emptying time. These factors may be the underlying disease, drugs used, psychological status, hormonal status, nutritional status and nutritional parameters, impaired exocrine function of the pancreas and complications related to the treatment method [11, 15, 16]. These multifactorial variables may be the reasons why our study and previous studies gave conflicting and different results in terms of complaints and findings mentioned above.

Gastritis is a quite common finding in CKD. In the literature, there are limited studies on the evaluation and comparison of gastritis findings in patients with CKD according to the treatment method. Wee et al. [17] found the prevalence of endoscopic gastroduodenitis to be 49% in a series of 322 patients under HD and PD treatment, that they underwent endoscopy. In the same study, they detected gastritis histologically in 52% of the cases in 260 patients who underwent endoscopic biopsy. In the same study, more gastritis was observed in the PD group than in the HD group. Misra et al. [18] found that GIS abnormalities were more common in patients with CKD in a study that they evaluated the endoscopic findings in patients with CKD and compared them with normal individuals. In terms of gastritis prevalence, Usta et al. [19] determined the frequency of histological gastritis as 62.3% in dialysis patients, Fabian et al. [20] determined the frequency of histological gastritis was 71.5%, Al-Mueilo et al. [21] found the frequency of histological gastritis 51.9% in hemodialysis patients. In our study, the patients were evaluated symptomatically in terms of gastritis and compared according to the RRT modality. The results were evaluated as 70% in the PD group, 60% in the HD group and 55% in the predialysis group. The findings were in parallel with the findings of Wee et al. [17] and were statistically significantly higher in the PD group than in the HD and predialysis groups. Although our study was a symptomatic evaluation and was not supported endoscopically or histologically, the frequency of gastritis found in our study shows similar rates with other studies. This determined prevalence may also be useful in showing that the diagnosis of gastritis based on the complaints and histories of the patients is similar to the frequency of gastritis diagnosis made endoscopically and histologically. Our study shows a statistically significantly higher prevalence of gastritis in the PD group like the result of Wee et al. [17]. Further studies are needed to clarify this result and its underlying causes.

Regurgitation, heartburn, dyspeptic complaints, dry cough and burping are common symptoms of gastroesophageal reflux. It is stated that gastroesophageal reflux is more common in peritoneal dialysis patients than in the general population and hemodialysis patients. It is stated that the presence of excess dialysis fluid in the abdomen and high intraperitoneal pressure increase acid reflux from the stomach [22]. Dejardin et al. [23] evaluated the relationship between intraperitoneal pressure, intraperitoneal volume and GER in a study and found that intraperitoneal pressure had no effect on reflux formation. Holscher et al. [22] reported that GER symptoms are seen around 30% in the normal population. In our study, the prevalence of GER was 38% in the predialysis patient group, 34% in the HD group and 45% in the PD group. Although these rates were higher than the general population, there was no statistically significant difference between the groups compared. Our study may give an idea that peritoneal dialysis treatment does not significantly increase the incidence of GER in patients with CKD; but the effect of peritoneal dialysis treatment on GER, the relationship of intraperitoneal pressure and intraperitoneal volume with GER is a less studied topic in the literature and deserves further studies.

A few other mechanical complications thought to be caused by intraperitoneal pressure (IPP) are constipation, hiatal hernia and hemorrhoids [24]. It is stated that the prevalence of hemorrhoids is 4.4% in the normal population in the USA [25]. In our patients, hemorrhoids were detected at a prevalence of 21% in the predialysis group, 12% in the HD group and 24% in the PD group. The prevalence of hemorrhoids was determined to be higher than the general population in all stages and treatment types of CKD. In addition, hemorrhoids were statistically significantly higher in the PD group than in the HD group; this supports the thesis that increased intraperitoneal pressure together with chronic constipation in peritoneal dialysis increases the risk of hemorrhoids, but it is not a sufficient result alone. Again, there was no statistically significant difference between the PD group and the predialysis group. In our clinic, an anti-constipation diet is recommended for patients under PD treatment and these patients are regularly treated with anti-constipation medications. In fact, it is seen in the study that there is no difference between PD and other groups in terms of constipation complaints. This may be due to the diet habits of the patients and the constipation-solving drugs they use. There is a close relationship between chronic constipation and hemorrhoids. There are many factors that can cause chronic constipation in chronic kidney disease and these factors have not been fully elucidated [16]. Many factors such as electrolyte disturbances like hypercalcemia, hypokalemia, uremic neuropathy seen in patients on long-term dialysis, increased colonic transit time, gastric hypomotility, drugs used, dietary habits, increased intraperitoneal pressure in peritoneal dialysis, treatment-related lifestyle and limitation of movement may be the cause of chronic constipation [11, 16, 24, 26–28]. The prevalence of constipation is reported to be 2–27% in Western Societies [29]. The results of our study show that the prevalence of constipation in CKD is higher than in the general population with a frequency of 32% in predialysis, 28% in HD and 35% in PD. There was no statistically significant difference between the groups. Although the prevalence of hemorrhoids was higher in the PD group, the lack of difference in the prevalence of constipation suggests that different factors such as IPP, which may be a factor in the formation of hemorrhoids in peritoneal dialysis, should be evaluated further.

The fact that the design of our study was in the form of a questionnaire and it included the subjective complaints of the patients may be misleading in determining the frequency. Although the results of some patients’ endoscopy, colonoscopy and abdominal imaging methods were included in the study, this does not cover the entire cohort. In terms of hiatal hernia, a statistically significant difference is observed in the HD group compared to the PD and predialysis groups. In terms of esophagitis, a statistically significantly higher frequency is seen in the HD group than in the predialysis group. However, in our study, the number of endoscopic cases was insufficient, and the distribution of endoscopy between groups was uneven. If the results of imaging examinations of all patients were included in the study, it would have been possible to reach a clearer judgment in these respects. However, evaluation and comparison of patients’ complaints with other findings in the study are important in terms of demonstrating gastrointestinal system involvement in CKD. Again, the results of our study should be supported by studies designed and evaluated with social averages and control groups without CKD.

## Conclusion

As a result; GIS symptoms and signs are quite common in patients with CKD. The majority of patients have more than one gastrointestinal symptom. In addition, when evaluated in terms of frequency, some of the GI symptoms and diseases vary according to the renal replacement therapy method, while some do not. Further studies are needed to evaluate physiopathological changes in the gastrointestinal system in CKD, the effects of these changes on GIS symptoms and signs, and the effects of symptoms and diseases on quality of life, morbidity and mortality.

Main Points:

- Gastrointestinal complaints and findings are common in CKD patients
- Dyspepsia, gastritis, nausea, anorexia, vomiting, gastroesophageal reflux findings, hemorrhoids and constipation are the findings often seen in CKD.
- Knowing the frequency of gastrointestinal symptoms, signs and diseases that may occur in CKD patients is valuable for early diagnosis and treatment of GIS disorders.
- Knowing the gastrointestinal symptoms, signs and diseases that may vary according to the treatment modality in CKD patients may help in the choice of renal replacement therapy model.

## Supplementary Information

**Additional file 1.**

## Data Availability

Available in supplementary materials.
